# Hybrid bright-field and hologram imaging of cell dynamics

**DOI:** 10.1038/srep33750

**Published:** 2016-09-19

**Authors:** Hyeokjun Byeon, Jaehyun Lee, Junsang Doh, Sang Joon Lee

**Affiliations:** 1 Center for Biofluid and Biomimic Research, Department of Mechanical Engineering, Pohang University of Science and Technology (POSTECH), Pohang, 790-784, South Korea; 2 School of Interdisciplinary Bioscience and Bioengineering (I-Bio), Pohang University of Science and Technology (POSTECH), Pohang, 790-784, South Korea

## Abstract

Volumetric observation is essential for understanding the details of complex biological phenomena. In this study, a bright-field microscope, which provides information on a specific 2D plane, and a holographic microscope, which provides information spread over 3D volumes, are integrated to acquire two complementary images simultaneously. The developed system was successfully applied to capture distinct T-cell adhesion dynamics on inflamed endothelial layers, including capture, rolling, crawling, transendothelial migration, and subendothelial migration.

To understand dynamic behaviors of biological cells, their morphological and 3D positional information are essential. Over the past decades, a variety of imaging techniques have been developed by using various microscopes composed of compound lenses, which enable high spatial resolution at the cost of shallow depth of focus. Scanning methods were developed to overcome the shallow depth of focus^1^,^2^,^3^. However, fast dynamic motions are rarely captured by scanning methods because of the scanning time; only slow dynamics or structural variations are acquired. Although other modalities have been employed to investigate biological cells^4^,^5^,^6^, their system configurations are complicated and usually require a fluorescence imaging method. When the dynamics of cells are spread over a 3D volume, researchers typically separate the overall dynamics into several parts, which falls within the depth of focus, and then investigate them part by part.

Holography, first introduced by Gabor^7^ in 1948, has received much attention because its volumetric recording and reconstruction enable to get information from various reconstruction planes. When a test object is located in the middle of a coherent laser beam, the beam diffracted by the object interferes with the remaining (undiffracted) beam, creating an interference pattern containing 3D volumetric information of the object. This interference pattern is called as a ‘hologram’. Numerical reconstruction of a hologram generates images at different planes by using the equation of light propagation. The reconstructed images contain information about complex light intensity and phase at each reconstructed plane. This information has been used for measuring 3D characteristics of the object, such as shape, size, position, etc.

Researchers have continuously improved the holography technique to adapt it to microscale objects. Digital holographic microscopy (DHM) was developed by combining the merits of optical microscopy and digital holography. This DHM can overcome the pixel size limitation which limits the resolving power of digital holography due to relatively large size pixel of CCD camera. Thus, 3D volumetric information about a microscopic sample could be obtained^8^. This development triggered a variety of new applications^9^,^10^,^11^,^12^. Among those applications, DHM has been considered an ideal technique for investigating biological samples because it does not require any labeling process^13^,^14^,^15^,^16^,^17^. In addition, compared with other microscopy techniques, in-line DHM has a relatively simple optical configuration with a fast reconstruction speed. Because the signal obtained from thin biological samples is weak, however, it is easily lost in the hologram. Therefore, conventional in-line DHM has been utilized for investigating the 3D dynamics of thick biological samples^18^,^19^,^20^,^21^.

In this study, a new optical system was developed to overcome the limitations of conventional optical microscopy and in-line DHM. By integrating the two techniques to obtain bright-field (BF) and hologram images together, the limitations of the two different techniques can be compensated. First, the use of the holography technique can solve the problem of information loss from cells that are not located in the focal plane caused by the shallow depth of focus of optical microscopy. Two-dimensional shape and 3D positional information about cells in a 3D volume can be obtained from a single hologram image. Second, the problem of a weak sample signal can be resolved by capturing bright-field images in the object plane where a test sample is located. In brief, the developed hybrid imaging system can simultaneously obtain information from the volumetric recorded hologram image and the BF image captured with a shallow depth of focus.

## Results and Discussions

**Figure 1:**
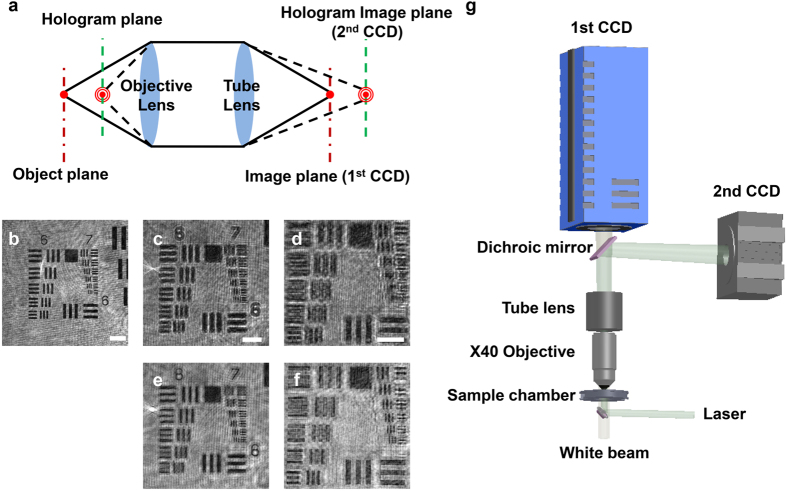
Principle and validations of the developed system. (**a**) Schematic of image plane locations according to different object plane locations. A sample in the object plane creates the corresponding image in the image plane. The image of the hologram is formed in the hologram image plane, which is far from tube lens relative to the image plane. (**b**) Focused image of a resolution test chart when the CCD was located in the image plane. (**c,d**) Holograms of the resolution test chart when the CCD was located at 100 mm and 300 mm from the image plane. (**e**,**f**) Reconstructed hologram images of the resolution test chart holograms **c**,**d** at z = 66 μm, 141 μm, respectively. Scale bar, 40 μm. (**g**) Schematic of the BF-hologram imaging system. The green laser beam and white beam pass through the sample chamber. The beams pass through the sample was magnified by the microscope objective and tube lens. The beams are split by a dichroic mirror. Only the green beam (hologram) reaches the second CCD; the other beam (bright-field image) reaches the first CCD. The schematic was illustrated by the authors using SolidWorks software (Dassault Systèmes SolidWorks Corp., USA).

Unlike BF images, holograms are usually generated above the object plane where test samples are located. As shown in Fig. 1a, when an object is located at the exact working distance away from the microscope objective, a magnified BF image of the object is shown at the original image plane. By contrast, in the case of a hologram, because hologram is located within the working distance of the microscope objective, the hologram image is located away from the original image plane. Therefore, both BF and hologram images can be obtained simultaneously by using two CCD cameras located at different image planes.

The performance of the developed imaging system was validated by varying the location of the CCD camera with a fixed test object location. Using a microscope objective, a focused image of the USAF 1951 resolution test chart was obtained when the CCD was located 200 mm from the tube lens installed inside the microscope (Fig. 1b). By moving the CCD further away, holograms were captured at a higher magnification and the required reconstruction depth was increased (Fig. 1c,d). The higher magnification was caused by beam divergence beyond the tube lens. The angle of divergence was measured to be 1.64°, which is sufficiently small to ignore the aberration caused by numerical reconstruction conducted with a parallel beam assumption. When we tested other microscope objectives with different magnifications, the required reconstruction depth was changed. Supplementary Table S1 provides a brief summary of the test results obtained with different microscope objectives.

In this study, a white beam and a green laser beam were utilized to generate BF and hologram images, respectively. Both beams passed through the sample and microscope objective and were separated by a dichroic mirror. Two cameras were installed at different locations to provide different optical path lengths for the two beams (Fig. 1c). The difference in optical path length for the two cameras enabled us to capture two images: a BF image and a hologram image of test object.

**Figure 2:**
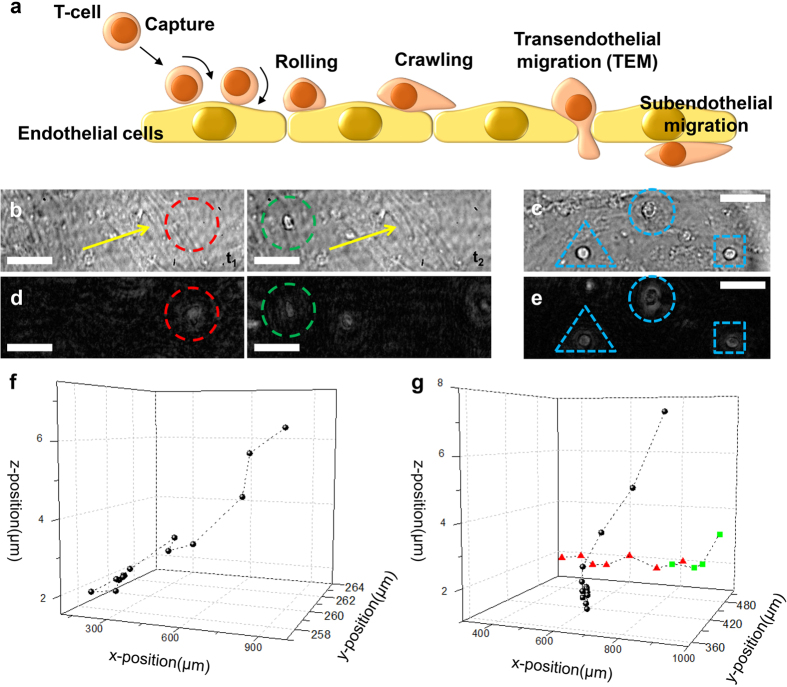
Captured 3D dynamics of T-cells on inflamed endothelium. (**a**) Schematic illustration of adhesion dynamics of T-cells on inflamed endothelium. (**b**) Bright-field images of T-cells and endothelial layers captured by consecutive recordings. Only endothelial cells (yellow arrows) and a T-cell adhered to the endothelial cells (green circle) are clearly observed; a flowing T-cell is not observed (red circle). (**c**) Bright-field image of T-cells at different status. (**d**) Reconstructed hologram image at the same location in bright-field image **b** at z = 139 μm (left) and at z = 143 μm (right). Reconstructed images from a T-cell attached to the endothelial cell (green circle) and a flowing T-cell (red circle) are captured. (**e**) Reconstructed hologram image at the same location in bright-field image **c** at z = 143 μm. The 2D shapes of T-cells are well resolved. Scale bars, 20 μm. (**f**) Trajectories of a flowing T-cell in 16 consecutive holograms. A T-cell gradually approaches the endothelial layer. (**g**) Trajectories of T-cells at different stages of adhesion dynamics.

The developed system was applied to investigate the dynamic motions of T-cells, a subset of immune cells, on endothelial cell layers^22^,^23^ (Fig. 2a). T-cells in the blood stream infiltrate inflamed tissues by executing a series of adhesion dynamic actions, including capture, rolling, crawling, transendothelial migration (TEM), and subendothelial migration. Analyzing T-cell dynamics requires 3D positional information about the T-cells because these movements occur over an entire 3D volume. In addition, information about the spatial distribution of endothelial cells is essential to analyze T-cell movement. However, it has been difficult for previous methods to obtain both sets of data simultaneously due to the limited depth of focus and/or weak signal. Because the developed hybrid system obtained both BF and hologram images at the same time, two sets of data could be acquired without a time delay.

The spatial distribution of endothelial cells and T-cells on the endothelial cells were observed by BF imaging only when the image was slightly defocused from the endothelial cell layers (Fig. 2b, yellow arrow and green circle). Therefore, cells suspended in media could not be observed, although their movements are also a major part of T-cell dynamics. By contrast, hologram images provided not only the signal associated with T-cells attached on the endothelial cells but also that of flowing T-cells, which could not be observed in the BF image (Fig. 2b, red circle). The reconstructed hologram image provided 2D shape and 3D positional information about T-cells at different status (Fig. 2d–e). It was possible to capture the shape of flowing T-cells in the media, which was impossible to observe in BF images (Fig. 2d, red circle). In addition, the shape of the attached T-cells was clearly observed and matched well with the shape shown in the BF image (Fig. 2d, green circle). Typical BF image, hologram and reconstructed images at different planes are shown in Supplementary Fig. S1. The 3D positional information enabled the 3D tracking of the motions of T-cells during the early stages of adhesion dynamics (Fig. 2f,g). Initially, T-cells in bulk flow approached endothelial layers (Fig. 2f), were captured on the endothelial layers (black dots in Fig. 2g), and began rolling on the endothelial layers (green squares and red triangles in Fig. 2g).

The developed hybrid system was also used to get image of T-cells during the late stages of adhesion dynamics, which are characterized by motions such as crawling and subendothelial migration. T-cells on endothelial layers crawl substantial distances to find sites to perform TEM (Supplementary Video S1). Because the spatial distribution of endothelial cells is important in this step, the information about the endothelial cells captured by BF images is necessary. The simultaneously obtained BF image provided this information while reconstruction of hologram images provided 3D positional information. After performing TEM, T-cells migrate underneath endothelial layers (Supplementary Video S2). T-cells underneath the endothelial layers were difficult to be distinguished from endothelial cells due to the weak signal in BF imaging (Supplementary Fig. S2). Similarly, the hologram signal from the T-cells underneath endothelial layers was too weak to be distinguished in the reconstruction of one hologram itself (Supplementary Fig. S2). To overcome this issue, the background subtraction method was adopted. Consecutive hologram images were averaged to obtain a background image. Because the timescale of T-cell motion was much faster than that of the changes in the endothelial cells^24^, only the hologram signal of moving T-cells could be acquired. Thus, the shape and 3D positional information of T-cells underneath endothelial layers (Supplementary Fig. S2) and T-cells undergoing subendothelial migration (Supplementary Video S2) could be obtained.

The developed hybrid system can acquire both BF and hologram images simultaneously without any labeling or additional treatment. Therefore, without damaging biological cells, the system can acquire information from a specific 2D plane and from the entire volume. Two-dimensional shape and 3D positional information are obtained from the reconstruction of holograms. In recording procedure of holograms, the minimal size of a test object depends on material properties of the test object. For biological cells, signal intensity is determined by the material filling the cells. If the biological cell is filled with dense, light-absorbing materials, the signal is strong. On the contrary, if the cell is filled with light, medium-like materials, its signal is weak and the minimum size of the cell becomes bigger. Therefore, it is difficult to specify the minimum size of test objects for the proposed system. In addition, the weak signal from thin biological cells, which are easily lost in the hologram recording procedure, can be obtained by capturing a BF image of a specific plane. The advantages of the hybrid system developed in this study were demonstrated by imaging the dynamic behaviors of T-cells.

The difference in magnification in two image planes implies that the field of view varies if the sizes of the two CCD cameras used for hologram and BF imaging are the same. To resolve this problem, the full size of the second CCD used to capture hologram images should be larger than that of the first CCD used for BF imaging. In this study, the size of the second CCD was nearly twice larger than that of the first one. In addition, since the diffraction pattern (hologram) of the object is generated at a location away from the object plane, the image plane should be moved to capture the focused hologram image instead of blurred image of the object. Therefore, if telecentric optical system which provides blurred images at constant magnification is applied, hologram with different magnification would appear. When the difference in field of view is irrelevant, only one CCD is required to obtain both BF and hologram images nearly at the same time. With minor modifications in the experimental setup (Supplementary Method and Supplementary Fig. S3), BF images, hologram images at different magnifications were obtained by using one CCD camera with a 50 ms delay (Supplementary Fig. S4).

The analytical determination of the distance between the two CCD cameras is difficult because manufacturers of microscope objectives utilize complicated compound lenses rather than simple thin lenses. Therefore, preliminary experiments are required to validate and establish the developed imaging system. By changing the location of the CCDs, the magnification and the defocusing depth can be flexibly changed. As the magnification of the microscope objectives increases, a longer distance is required to achieve the same defocusing depth. Therefore, the tradeoff between the defocusing depth and the magnification should be considered in the design of the optical configuration of the system.

The current system utilizes BF images. Because a BF image is the basic result that can be obtained by an optical microscope, further improvement can be made by applying different optical techniques, such as fluorescence microscopy. By adding related optical components, fluorescence images or fluorescent holograms can be obtained without disturbing other images^25^,^26^.

## Method

### Sample preparation

DO11.10 T-cell receptor transgenic mice were purchased from Jackson Laboratories and bred in the animal care facility of the POSTECH Biotech Center (PBC). All experimental procedures and protocols, including mice treatments, were performed in accordance with the guidelines approved by the Institutional Animal Care and Use Committee at PBC. The experiments were conducted in accordance with the approved guidelines. Cells harvested from lymph nodes and spleens of DO11.10 mice were cultured in RPMI 1640 (Gibco, USA) containing 10% FBS (Gibco), 1% penicillin–streptomycin (Invitrogen), and 0.05 uM beta-mercaptoethanol (Sigma, USA) in the presence of 1 μg/ml of OVA323–339 peptide (ISQAVHAAHAEINEAGR, Peptron, Inc. Korea). After 2 days, 1–2 U/ml (5 ng/ml) of IL-2 (Peprotech, USA) was added. T-cell blasts were cultured for another 3 days and used for experiments after removing dead cells using Histopaque (Sigma). The diameter of the T-cells tested in this study was the range of 7~9 μm.

Coverglasses (Marienfeld) were cleaned by treating with air plasma (200–500 w, Femto Science, Korea) for 1 min, coated with 0.1% gelatin (Sigma) in PBS for 30 min at 37 °C, and washed with PBS. Approximately 1.5 × 10^5^ bEnd.3 cells were seeded on the gelatin-coated coverglasses and cultured in DMEM containing 10% FBS and 1% penicillin–streptomycin for 48 h to form a confluent monolayer. Then, bEnd.3 cell monolayers were activated by treating with TNF-α (10 ng/ml) for 4 h prior to flow experiments.

The test channels consisted of two coverglasses and a PDMS wall. The PDMS wall was fabricated as follows. FEP tubes with a diameter of 200 μm were inserted in a mixture of PDMS prepolymer (Sylgard 184; Dow Corning, USA) and a curing reagent at a mass ratio of 10:1. All trapped air bubbles were removed in a vacuum chamber for 1 h. Then, they were cured at 80 °C for 6 h. The cured PDMS was punched with a custom-made cutting device to make the channel wall rectangular. The PDMS was covered with two coverglasses: one with an endothelial cell layer and the other with no treatment. The PDMS channel and coverglasses were securely held by a magnetic chamber (Chamlide CF-T, Live Cell Instrument, Korea), as depicted in Supplementary Fig. S5. The fabricated PDMS channel was rectangular in cross section with a width of 2 mm and height of 0.5 mm. The FEP tube was connected to a syringe pump (Longer Precision Pump, China) and inline heaters (Live Cell Instrument, Korea) to control flow conditions and to maintain the temperature of the media at 37 °C. Initially, T-cells suspended in the growth medium at a concentration of 2 × 10^6 ^cells/ml were injected at 0.25 dyne/cm^2^ for 10 min to accumulate T-cells on the endothelial cell layer. Then, shear stress was gradually increased to 2 dyne/cm^2^ with growth medium alone to observe full adhesion cascades of T-cells^27^.

### Experimental setup

A solid-state laser (λ = 532 nm, 100 mW, CrystalLaser, USA) and a halogen lamp were used as the light sources for hologram and BF imaging, respectively. After the beams passed through the sample chamber, they passed through a water-immersion microscope objective (40x, NA = 0.8, Nikon, Japan) to magnify the images. The original tube lens inside a microscope (Eclipse 50i, Nikon, Japan) made the beam less divergent. A dichroic mirror that reflects green light was installed after the tube lens to divide the beam into two beams for BF and hologram imaging. Thus, only BF image was acquired in the first CCD (PCO.1200hs, PCO, Germany). BF images were consecutively recorded at a frame rate of 1fps with an exposure time of 50 ms. In this study, slightly defocused BF images were utilized (5 μm above the focus plane). The spatial distribution and the shape of cells were clearly distinguished in the slightly defocused image rather than in the exactly focused image. This phenomenon is caused by transparency of endothelial cells. For the case of BF imaging, it is difficult to distinguish transparent cells from background. To secure experimental space, the first CCD was located 220 mm away from the tube lens. The second CCD (Ultima-APX, Photron, Japan) was located 580 mm away from the tube lens to capture hologram images. Hologram images were recorded at the same frame rate used to capture BF images with an exposure time of 33 us. Two CCD cameras were connected to a delay generator to start their exposure at the same time for capturing both images.

### Reconstruction and 3D positioning

Before numerical reconstruction of holograms, the background image was subtracted from raw holograms to extract fringe patterns generated from test cells by excluding noise caused by optical components and their misalignment. In this study, more than 500 consecutive hologram images were averaged to make the background image.

Hologram images were numerically reconstructed by adopting the Fresnel-Kirchhoff diffraction formula. The formula consists of convolution of the hologram function *h*(*x, y*) and the diffraction kernel *g*(*ξ, η*). The following convolution theorem was employed in the reconstruction process:


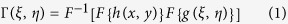


where *F* and *F*^*−1*^ represent the Fourier transform and its inverse, respectively. *x, y* and *ξ, η* denote the spatial coordinates in the hologram and the reconstruction planes, respectively. The angular spectrum method was adopted to obtain the Fourier transform of the diffraction kernel *g*(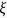, 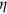) as follows:


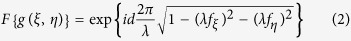


where *f*_*ξ*_ and *f*_*η*_ denote the spectral coordinates, *d* is the distance between the hologram and the reconstruction planes, and *λ* is the wavelength of laser light. The Fourier transform was numerically computed using fast Fourier transform. Details regarding the reconstruction procedures adopted in this study can be found in previous studies^11^,^28^.

After holograms were numerically reconstructed with a depth-wise spacing of 1 μm, reconstructed hologram images were projected onto a 2D projection image. The in-plane (*x, y*) position was determined by locating the point of local maximum intensity in the projection image. After obtaining the in-plane (*x, y*) position of a cell, a segmented image was generated around the peak (*x, y*) position. The z-position of the cell was determined by applying an autofocus function to the segmented image. The Tamura coefficient^29^, TC, was determined based on the intensity distribution using the following equation:


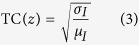


where *σ*_*I*_ and *μ*_*I*_ denote the standard deviation and the mean value of light intensity distribution in the reconstructed segment image. The reconstructed segment images and TC value of T-cell along the depth-wise direction are shown in Supplementary Fig. S6. The reconstruction depth with the minimum TC value was determined as the z-position of the cell.

## Additional Information

**How to cite this article**: Byeon, H. *et al*. Hybrid bright-field and hologram imaging of cell dynamics. *Sci. Rep.* **6**, 33750; doi: 10.1038/srep33750 (2016).

## Supplementary Material

Supplementary Information

Supplementary Video 1

Supplementary Video 2
