# Supplementary material for: Hybrid bright-field and hologram imaging of cell dynamics
Source: Sci Rep. 2016 Sep 19;6:33750. doi: 10.1038/srep33750 (PMC5027394; doi:10.1038/srep33750)
Supplement: Supplementary Information [file srep33750-s1.pdf]

Supplementary information

Hybrid bright-field and hologram imaging of cell dynamics

Hyeokjun Byeon[a], Jaehyun Lee[b], Junsang Doh[a,b,**], and Sang Joon Lee[a,b,*]

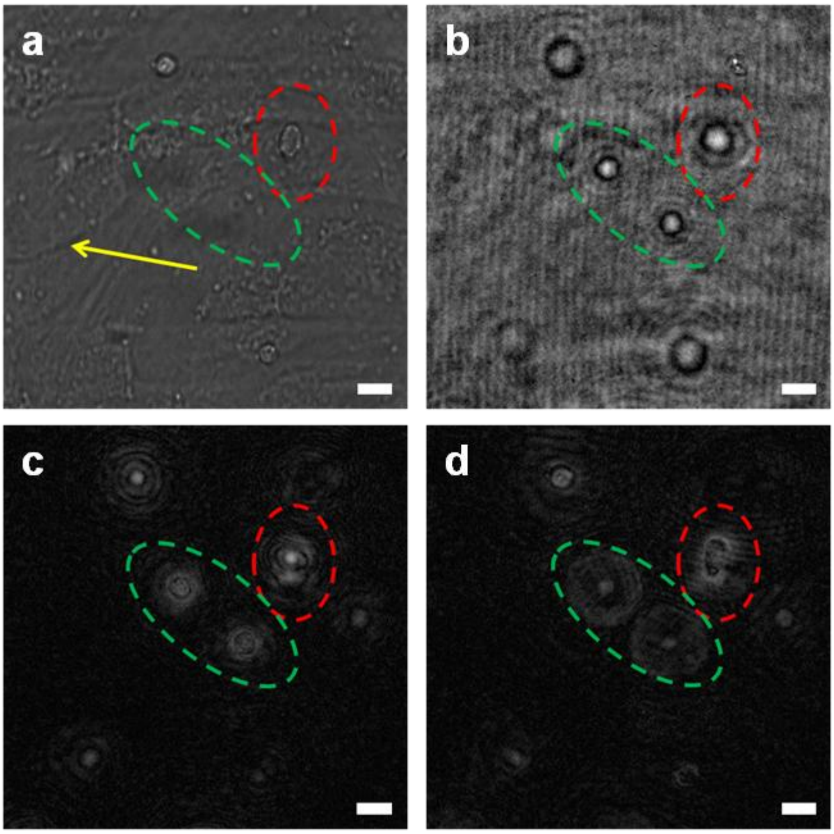

Supplementary Figure S1 (**a**) A typical bright field image of T-cells and endothelial cells. T-cell in the focus (red ellipse) and endothelial cells (yellow arrow) are observed. No cell signal appears in the middle of the image (green ellipse). (**b**) Hologram image at the same location of the bright field image **a**. Hologram images of T-cell attached to the endothelial cell (red ellipse) and flowing T-cells (green ellipse) were simultaneously captured. (**c**) Reconstructed hologram image at z=68μm. Spherical shape of flowing T-cells is well observed (green ellipse). (**d**) Reconstructed hologram image at z=138μm also clearly shows the shape of the attached T-cell (red ellipse). Scale bar, 10μm.

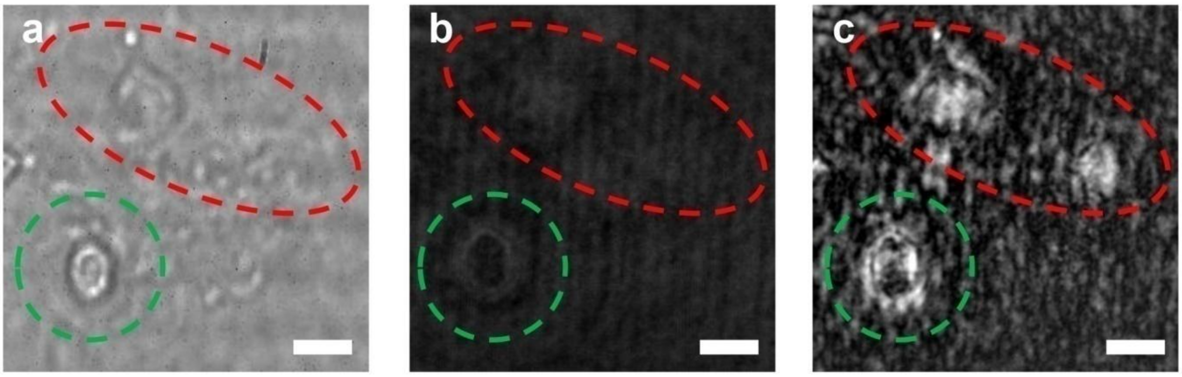

Supplementary Figure S2 Bright-field and hologram image of T-cells. (**a**) Bright-field image of T-cells on the endothelial layer (green circle) or underneath the endothelial layer (red ellipse). (**b**) Reconstructed hologram image without background subtraction. The signal from T-cells underneath the endothelial layer is difficult to distinguish (red ellipse) from that of the endothelial layer. (**c**) Reconstructed hologram image with background subtraction. The 2D shape of the T-cells underneath the endothelial layer is clearly observed (red ellipse). Scale bar, 10μm.

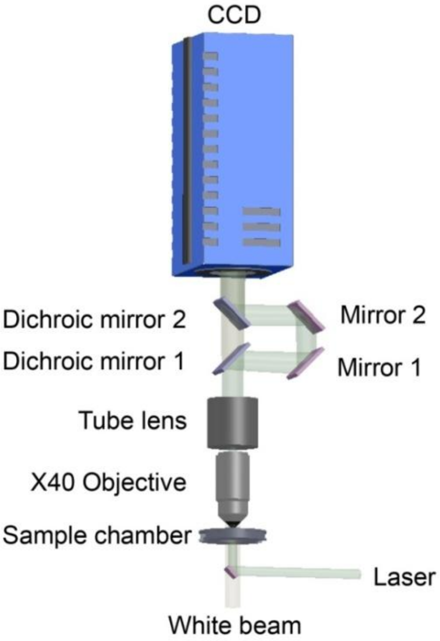

Supplementary Figure S3 Schematic of the BF-hologram imaging system composed of one CCD. The green and white beams pass through the sample chamber. The beams passed through the sample are magnified by a microscope objective. The beams are split by a dichroic mirror. The optical path of the green beam is lengthened by mirrors. The green beam reaches the CCD after passing the auxiliary optical path, whereas the other color beam directly reaches the CCD. The schematic was illustrated by the authors using SolidWorks software (Dassault Systèmes SolidWorks Corp., USA).

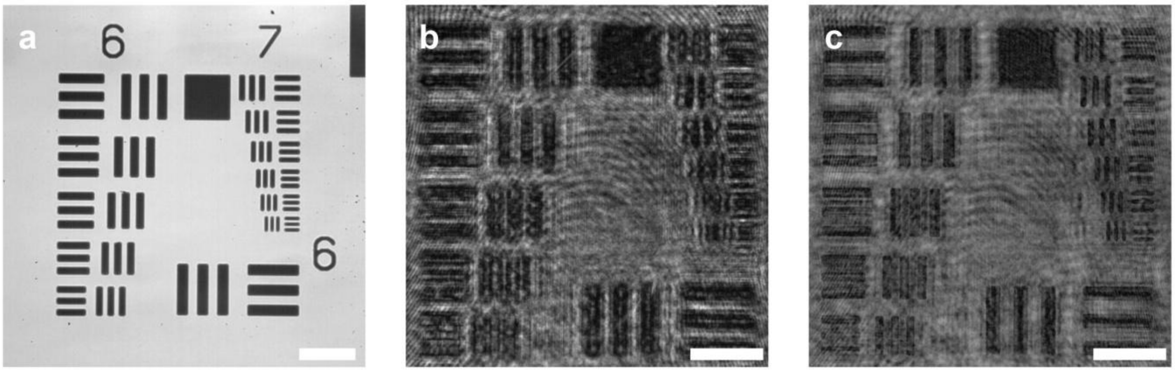

Supplementary Figure S4 Bright-field and hologram images of test chart with one CCD camera (**a-b**) Bright-field image and hologram captured by the BF-hologram imaging system with one CCD. (**c**) Reconstructed hologram image captured at a depth of z=177μm. Scale bar, 40μm.

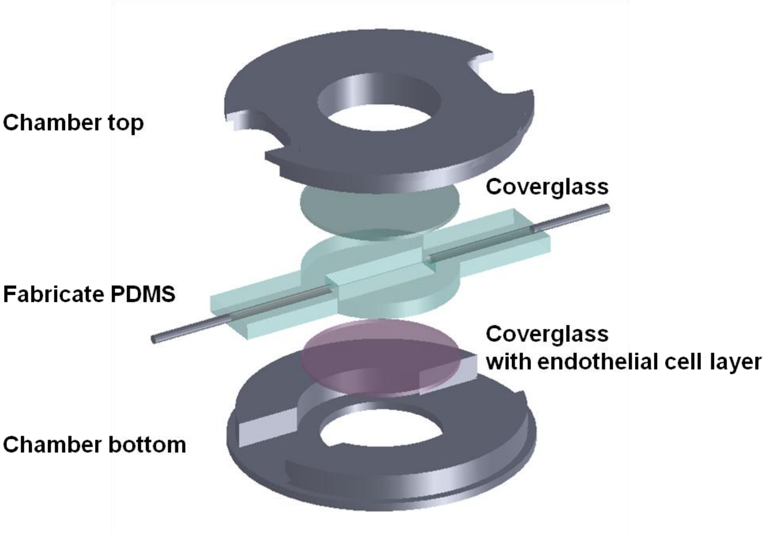

Supplementary Figure S5 Schematic of experimental chamber with a fabricated PDMS channel, a plain coverglass and a coverglass coated with an endothelial cell layer. The schematic was illustrated by the authors using SolidWorks software (Dassault Systèmes SolidWorks Corp., USA).

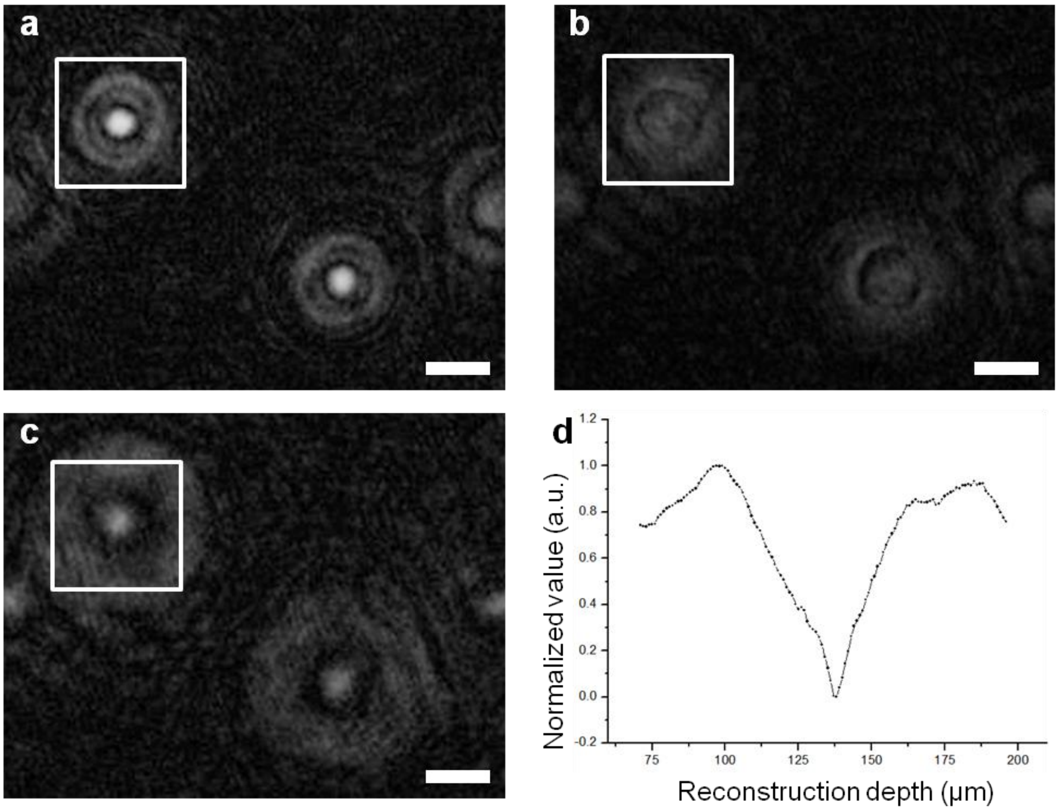

Supplementary Figure S6 Typical reconstructed images of T-cell (**a**) at z = 97μm (**b**) at z = 138μm (**c**) at z = 179μm **(d)** Variation of autofocus function in the segmented image (white square in (a-c)). Scale bar, 10μm.

Supplementary Table S1:Variation in reconstruction depth according to distance between CCDs and microscope objective used

Distance (mm)	Microscope Objectives		
	20X (NA=0.5)	40X (NA=0.8)	60X (NA=1.0)
0	0	0	0
100	275	66	29
200	474	108	48
300	606	141	64
400	685	167	76

*The unit of reconstruction depth is μm

The measured reconstruction depth (μm) varied according to the microscope objective used and the distance between the CCD and original focal plane of the tube lens.

Supplementary method

A solid-state laser (λ = 532 nm, 100 mW, CrystalLaser, USA) and a halogen lamp were employed as light sources. After the beams passed through the sample chamber, a water-immersion microscope objective (40X, Nikon, Japan) magnified the sample images. A dichroic mirror that reflects the green beam was installed to divide the beams into two beams for BF and hologram imaging. The green beam was reflected by mirrors, and another dichroic mirror was installed to make the beam path longer before the beam reached the CCD (Supplementary Fig. 2). Thus, the optical path length of the green beam was 190mm longer than that of the white beam excluding the green light. Interval blocking of the laser beam allowed for BF and hologram images to be obtained sequentially by the CCD camera (PCO.1200hs, PCO, Germany). The exposure times for BF and hologram imaging were 50ms and 0.5ms, respectively.

Supplementary Video captions

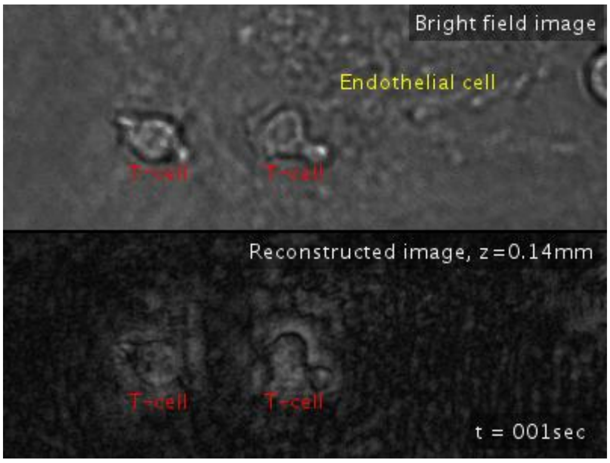

Supplementary Video S1: Crawling T-cells on endothelial cells

Crawling motion of T-cells on endothelial cells are well observed in both bright-field (top) and reconstructed hologram images at z=140µm (bottom). The endothelial cells are only observed in bright-field images. The depth-wise location of a cell was determined by searching the reconstruction depth, which yields the image with the lowest auto focus function value.

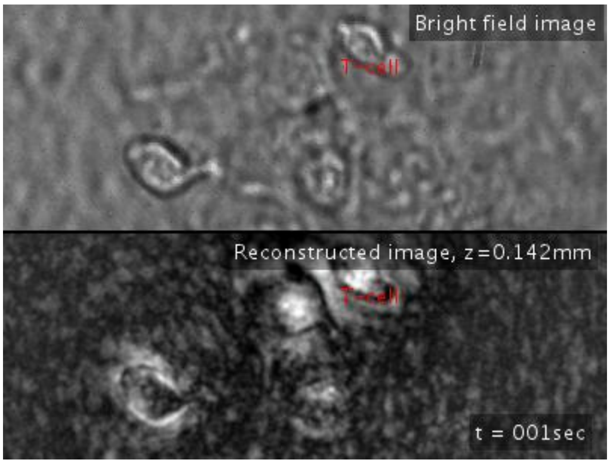

Supplementary Video S2: Crawling and TEM of T-cell with endothelial cell

The TEM image of T-cells is better observed in the reconstructed hologram image at a depth of z=142µm (bottom, after t=385sec) than in the bright-field image (top), where the T-cell underneath the endothelial layer after performing TEM is not distinguished from other cells. The shapes of the T-cells underneath the endothelial layer can be obtained only from the reconstructed hologram images.
